# Longitudinal, Quantitative, Multimodal MRI Evaluation of Patients With Intracerebral Hemorrhage Over the First Year

**DOI:** 10.3389/fneur.2021.764718

**Published:** 2021-11-30

**Authors:** Muhammad E. Haque, Seth B. Boren, Octavio D. Arevalo, Reshmi Gupta, Sarah George, Maria A. Parekh, Xiurong Zhao, Jaraslow Aronowski, Sean I. Savitz

**Affiliations:** ^1^Institute for Stroke and Cerebrovascular Diseases and Department of Neurology, Louisiana State University, Shreveport, LA, United States; ^2^Biostatistics, Epidemiology, and Research Design Component, Center for Clinical and Translational Sciences, Louisiana State University, Shreveport, LA, United States; ^3^Department of Radiology, McGovern Medical School, The University of Texas Health Science Center at Houston, Louisiana State University, Shreveport, LA, United States

**Keywords:** intracerebral hemorrhage stroke, quantitative susceptibility mapping (QSM), arterial spin labeling, diffusion tensor imaging, serial magnetic resonance imaging

## Abstract

In most patients with intracerebral hemorrhage (ICH), the hematoma and perihematomal area decrease over the subsequent months but patients continue to exhibit neurological impairments. In this serial imaging study, we characterized microstructural and neurophysiological changes in the ICH-affected brain tissues and collected the National Institute of Health Stroke Scale (NIHSS) and modified Rankin Score (mRS), two clinical stroke scale scores. Twelve ICH patients were serially imaged on a 3T MRI at 1, 3, and 12 months (M) after injury. The hematoma and perihematomal volume masks were created and segmented using FLAIR imaging at 1 month which were applied to compute the susceptibilities (χ), fractional anisotropy (FA), mean diffusivity (MD), and cerebral blood flow (CBF) in the same tissues over time and in the matching contralesional tissues. At 3 M, there was a significant (*p* < 0.001) reduction in hematoma and perihematomal volumes. At 1 M, the χ, FA, and CBF were decreased in the perihematomal tissues as compared to the contralateral side, whereas MD increased. In the hematomal tissues, the χ increased whereas FA, MD, and CBF decreased as compared to the contralesional area at 1 M. Temporally, CBF in the hematoma and perihematomal tissues remained significantly (*p* < 0.05) lower compared with the contralesional areas whereas MD in the hematoma and χ in the perihematomal area increased. The NIHSS and mRS significantly correlated with hematoma and perihematomal volume but not with microstructural integrity. Our serial imaging studies provide new information on the long-term changes within the brain after ICH and our findings may have clinical significance that warrants future studies.

## Introduction

Intracerebral hemorrhage (ICH) accounts for about 10–15% of all strokes and is associated with high mortality (1, 2). ICH-induced death may occur within hours to days of the ictus (3, 4). ICH is characterized by two primary modes of injury: (1) mechanical stress due to rupture of brain blood vessels, physical compression of brain tissues, an increase in intracranial pressure, and midline shift; (2) high levels of iron concentration from hemoglobin and its breakdown products causing cytotoxic and vasogenic edema, iron toxicity, oxidative stress, protein, and DNA damage (5–11). ICH also causes neuroinflammation, which has been characterized by the presence of neutrophils, and macrophages/monocytes as well as microglial activation around the hemorrhagic foci (12–14). In the acute phase of ICH, hematoma and perihematomal volume is routinely monitored using computed tomography (CT) in the hospitalized setting but the long-term effects on tissue physiology such as cerebral blood flow (CBF) and microstructural integrity have received little attention (15, 16). While hematomas are absorbed over time and tissue is restored to partial or full normal-appearing (17), patients continue to exhibit hemiparesis and other neurological impairments. Recent advancements in non-invasive quantitative magnetic resonance imaging enable us to serially probe the integrity and physiological status of the hematomal and perihematomal tissues over the long term. In this pilot study, we aimed to longitudinally monitor changes in the iron concentration, microstructural integrity, and cerebral perfusion within the perihematomal and hematomal tissues using quantitative susceptibility mapping (QSM), diffusion tensor imaging (DTI), and arterial spin labeling (ASL), respectively. We selected QSM to measure iron induced susceptibilities over gold standard gradient echo (GRE) to eliminate acquisition parameter dependency and hematoma volume overestimation, well-documented phenomena (17). We selected DTI to evaluate the microstructural integrity of the tissues after hematoma resolution and ASL as a marker of tissue functional status. We hypothesize localized change in blood flow over time will only occur if tissues are viable and functional after hematoma and edema re-absorption. Our study provides detailed findings on the dynamic changes in a number of novel endpoints as an initial approach to understanding and characterizing the long-term evolution of ICH within the first year after injury.

## Methods and Procedures

### Patient Enrollment and Human Protection

Twelve patients with ICH admitted to the Memorial Hermann Hospital-Texas Medical Center between 2016 and 2020 participated in a longitudinal MR imaging study. The study was approved by the institutional review board of the University of Texas Health Sciences Center at Houston and by the Memorial Hermann Hospital Office of Research. Written informed consent was obtained after a thorough discussion with patients and family members. The inclusion criteria were all patients diagnosed with parenchymal ICH age 18–80 years, hematoma volume <100 cc, NIHSS 0–20. Patients with a brain tumor, traumatic brain injury, brain aneurysm, claustrophobia, and metal implantation were excluded.

### Neurological and Radiological Assessments

All participants underwent baseline and serial assessment of neurological deficits via the NIHSS (18) and mRS, a disability severity score (19, 20). The neurological assessments were correlated with the hematoma volume, perihematomal volume, and imaging matrices (χ, FA, MD, and CBF) within the hematoma and perihematomal ROI. Baseline images (CT or MRI) were obtained within 6–24 h of onset as part of the standard of care protocols without quantitative MRI. Follow-up imaging was obtained at 1 month (M), 3, and 12 M. The qualitative radiological assessment included serial changes in hematoma and perihematomal volume, acute presence of mass effect or midline shift, and development of Wallerian Degeneration (WD).

#### Imaging Sequences

Complete detail of these imaging methods can be found elsewhere, but are briefly summarized below.

*Quantitative susceptibility mapping (QSM)*, an emerging advanced image-processing algorithm of gradient echo (GRE) imaging, is highly sensitive and specific to magnetic susceptibility sources. Unlike GRE, QSM not only measures hematoma volume accurately, but also creates a clear delineation between paramagnetic substances (such as iron, ferritin, and hemosiderin) from diamagnetic substances (such as calcium, water, brain tissues) by positive and negative susceptibilities, respectively. The voxel intensity in QSM is linearly proportional to the source of susceptibilities (χ) and using an external calibration curve, these maps can be applied to compute iron concentrations within the hematoma and surrounding perihematomal area (21–23).

*Diffusion Tensor Imaging (DTI)* exploits both the magnitude and direction of water molecules diffusion as a tracer to probe tissue integrity. It can provide quantitative information *via* measuring scalar matrices known as fractional anisotropy (FA) and mean diffusivity (MD). On the FA scale between 0 (un-restricted or isotropic diffusion) and 1 (restricted or anisotropic diffusion), a decrease in FA indicates microstructural damage. The mean diffusivity can tag the magnitude of the water molecule diffusion coefficient, which changes in damaged tissues (24, 25).

*Arterial Spin Labeling (ASL)* measures cerebral perfusion by employing arterial blood as an endogenous tracer labeled with a radio frequency pulse. These labeled water molecules in the blood are allowed to enter the brain parenchyma and images are obtained at 1–2 s delay. Subsequently, images are also obtained without labeling, and perfusion contrast is calculated by subtracting the labeled and non-labeled images depicting the exchange of magnetization (26, 27).

### Image Acquisition

Serial images were obtained on a full body 3.0 T Philips Intera system that was later upgraded to Ingenia (Philips Medical Systems, Best, Netherland). Anatomical imaging included: 3D T1-weighted (TR/TE = 8.11/3.74 ms, imaging matrix = 256 × 256 × 180 mm^3^, slice thickness = 1 mm), and 3D fluid attenuated inversion recovery (FLAIR, TR/TE = 4800/129 ms, imaging matrix = 256 × 256 × 180 mm^3^, slice thickness = 1 mm). Quantitative imaging included: 3D quantitative susceptibility mapping (multiecho GRE, number of echo = 8, echo spacing = 6.93 ms, TR = 57.7 ms, first TE = 4.52 ms, voxel size = 0.75 x 0.75 x 1.0 mm^3^), diffusion-tensor imaging (TR/TE = 9.5 s/66 ms, b-value = 0, 1000 s/mm^2^, number of gradient direction = 21, matrix = 128 128, slice thickness = 3 mm), arterial spin label (PCASL, labeling duration/delay = 1.9 /2.0 sec, number of dynamics = 70, TR/TE = 4.8 s/15.8 ms, slice thickness/no slice = 5 mm, martix = 88 x 88 mm^2^).

### Image Processing

The DTI and ASL data were pre-processed using FSL (http://www.fmrib.ox.ac.uk/fsl). The QSM maps were computed using Susceptibility Tensor Imaging (STI) software (https://people.eecs.berkeley.edu/~chunlei.liu/software.html) to compute susceptibilities (χ). T1-weighted and FLAIR images were registered to FA, MD, CBF, and QSM quantitative maps.

### Lesion Volume Measurements and Region of Interest

**Figure 1:**
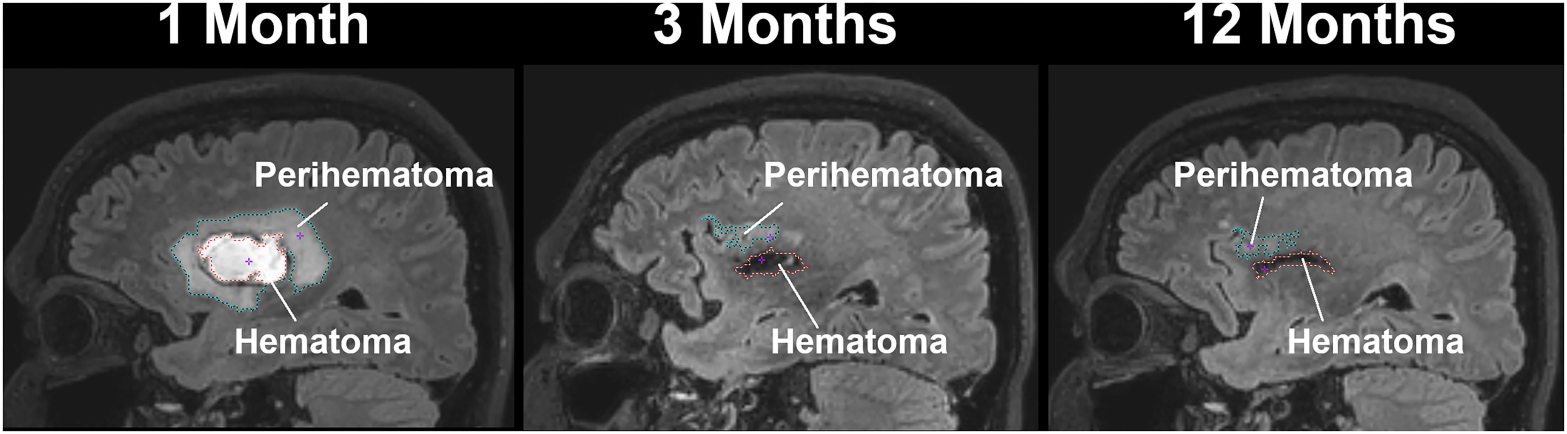
A sagittal view of a patient with intracerebral hemorrhage displaying temporal changes in a hematoma (red-dotted line) and perihematomal (cyan dotted line) volume.

A semi-automated seed growing algorithm using Analyze 12.0 (Analyze Direct Inc., KS, USA) software was used to delineate and compute hematoma and perihematomal volume on FLAIR images at all three-time points as shown in Figure 1. Briefly, a seed point was selected within the hematoma and signal intensity was threshold so neighboring pixels with similar signal intensity were added to the seed region to create a hematoma mask. The process was iterated several times and the hematoma mask was manually edited whenever necessary by including or removing pixels. The same process was repeated to delineate perihematomal mask. The hematoma and perihematomal masks that were delineated at 1 month were saved as ROI and registered to the QSM, FA, MD, and CBF maps to compute temporal imaging matrices in the same regions. The same ROI was also used for contralesional healthy tissues. We equated the perihematomal area with cerebral edema.

### Statistical Analysis

Standard descriptive statistics summarized participant characteristics at baseline. Categorical variables were calculated as frequencies and percentages. A quantile-quantile plot was used to determine whether response variables (hematoma and perihematomal volumes, magnetic susceptibility, fractional anisotropy, mean diffusivity, and CBF) were normally distributed and amenable to analysis with traditional parametric techniques. Linear mixed models with random intercept and unstructured covariance were used to determine the changes over time in the above-mentioned variables from baseline. The statistical significance level was set at α = 0.05 (i.e., the probability of rejecting the null hypothesis when it is true is set at 0.05) with 95% confidence intervals for all the model parameters. Bonferroni adjustment for multiple testing was used for all *post hoc* comparisons. All analyses were conducted with SAS statistical software version 9.4 (SAS Institute Inc., Cary, NC).

## Results

### Demographics and Clinical Information

Imaging data from a total of 12 ICH patients (35 scans) were used in this analysis. All participants completed all the visits except one patient who missed the last visit. There were eight men and four women with an average age of 54.4 ± 16.6 years with no previous stroke. All patients had unilateral hemorrhage with six patients in the left and the other six in the right hemisphere. The median NIHSS on admission was four (IQR 2–9.5) and decreased to 0 (IQR 0–2) over 1 year. The median mRS was 2.5 (IQR 1–3.25) at 1 M and 1.5(IQR 0.5–2) at 12 M. Only one patient underwent acute hemicraniectomy due to expansion of edema. All participants had hypertensive bleeding except P12 who had a venous vascular malformation and P09 who had a cerebral venous thrombosis. P11 might have had bleeding due to anticoagulation. None of the patients had bleeding due to cerebral amyloid angiopathy or metastasis. All patients exhibited variable degrees of mass effects and four patients had midline shifts in the acute phase. Four patients showed signs of Wallerian Degeneration by the end of the study. The demographics and qualitative radiological assessment, and clinical scores are summarized in Table 1.

**Table 1:** Patient demographics and clinical assessment.

**PID**	**Age/Gender**	**Lesion side**	**Lesion location**	**NIHSS acute/12 M**	**mRS 1 M/12 M**	**Edema 1 M/12 M**	**Hematoma 1M/12M**	**Midline shift (acute)**	**WD (01 M)**	**WD (12 M)**
						**Volume (cc)**				
P01	60/M	R	Insula, CST, BG	5/NA	0/NA	20.7/NA	0.54/NA	N	N	N
P02	69/M	L	Precuneus	0/0	1/0	28.6/4.45	21.2/0.37	N	N	N
P03	64/M	L	BG, CST	4/3	3/2	2.0/2.14	5.8/0	N	N	Y
P04	67/F	L	BG, CST	13/2	4/3	2.3/1.85	5.1/0	N	N	N
P05	54/M	R	Thalamus, BG	3/0	1/1	7.2/1.01	6.6/0.15	N	N	N
P06	52/M	L	CR, putamen, Insula	4/1	3/2	17.3/1.44	6.6/0	Y	N	Y
P07	33/M	R	Fronto-parietal, BG, Internal Capsule	14/3	4/2	12.7/27.1	55.5/0	Y	N	Y
P08	35/M	R	Insula, CST, BG	15/2	4/2	49.9/6.71	62.4/0.16	Y	N	Y
P09	29/F	L	Temporal lobe	2/0	2/0	71.4/4.35	27.9/0	Y	N	N
P10	72/F	R	BG, CR, Thalamus	2/0	0/1	4.2/0.59	1.3/0	N	N	N
P11	78/F	L	Temporo-occipital	6/0	1/2	5.6/1.34	4.8/0	N	N	N
P12	40/M	R	Vermian Cavernoma	0/0	2/0	2.1/0.66	0.86/0	N	N	N

*NIHSS, National Institute of Stroke Scale; WD, Wallerian Degeneration; CST, Corticospinal Tracts; CR, Corona Radiata; BG, Basal Ganglia*.

### Hematoma and Perihematoma Volume

**Figure 2:**
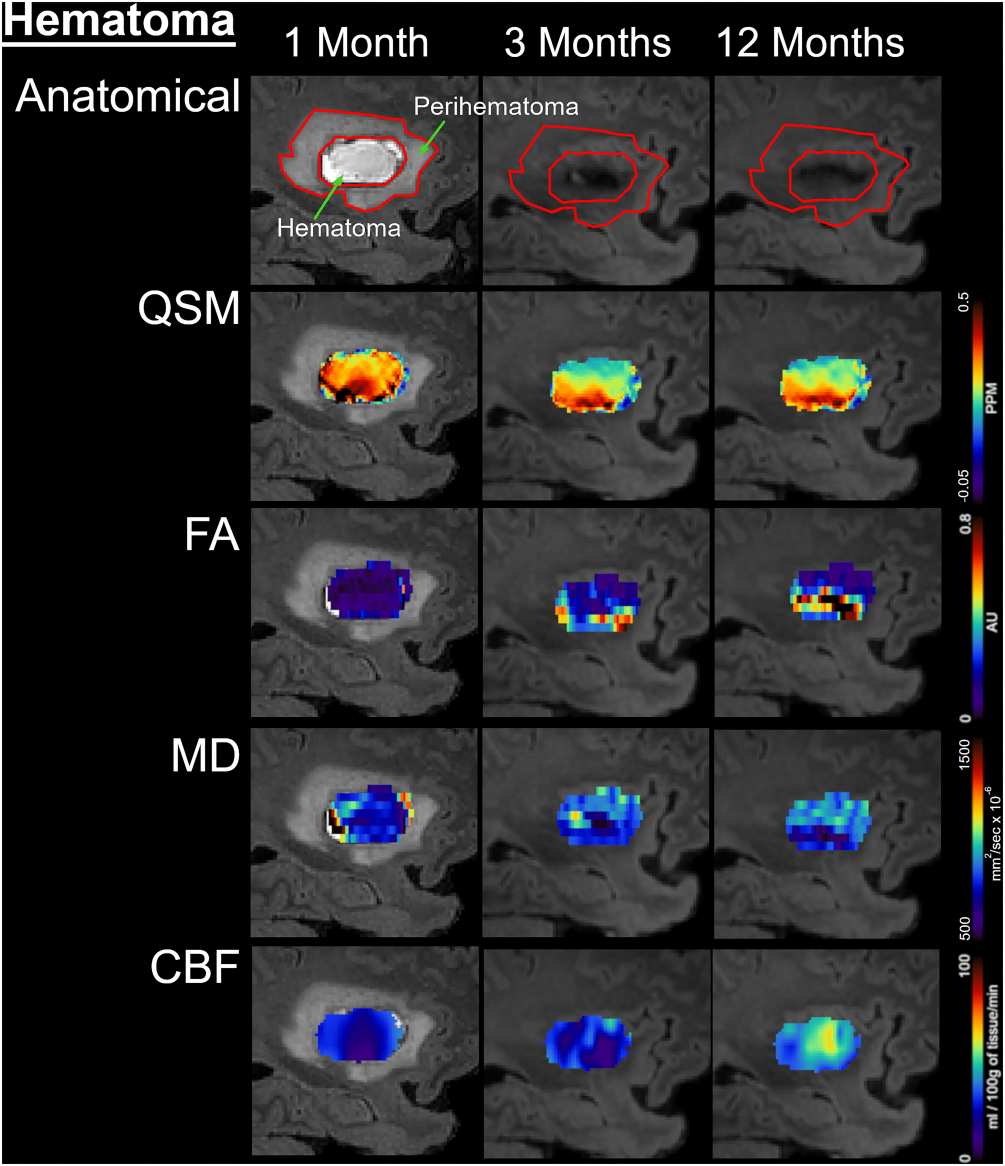
Temporal changes in the hematomal tissues are shown on a fluid-attenuated inversion recovery (FLAIR) by color-coded voxel by voxel quantitative maps in a patient. The rows are illustrating the imaging measurements whereas columns are showing changes at 1, 3, and 12 M after onset. In the top row, the first image outlines the perihematomal and hematoma regions that were used as an ROI to measure imaging matrices over time. The other two images on the same row are showing the decrease in hematoma volume with a hypo-intense hemosiderin region at 3 and 12 M. The rows 2–5 display QSM maps, FA, MD, and CBF in the hematomal tissues over time, respectively.

**Figure 3:**
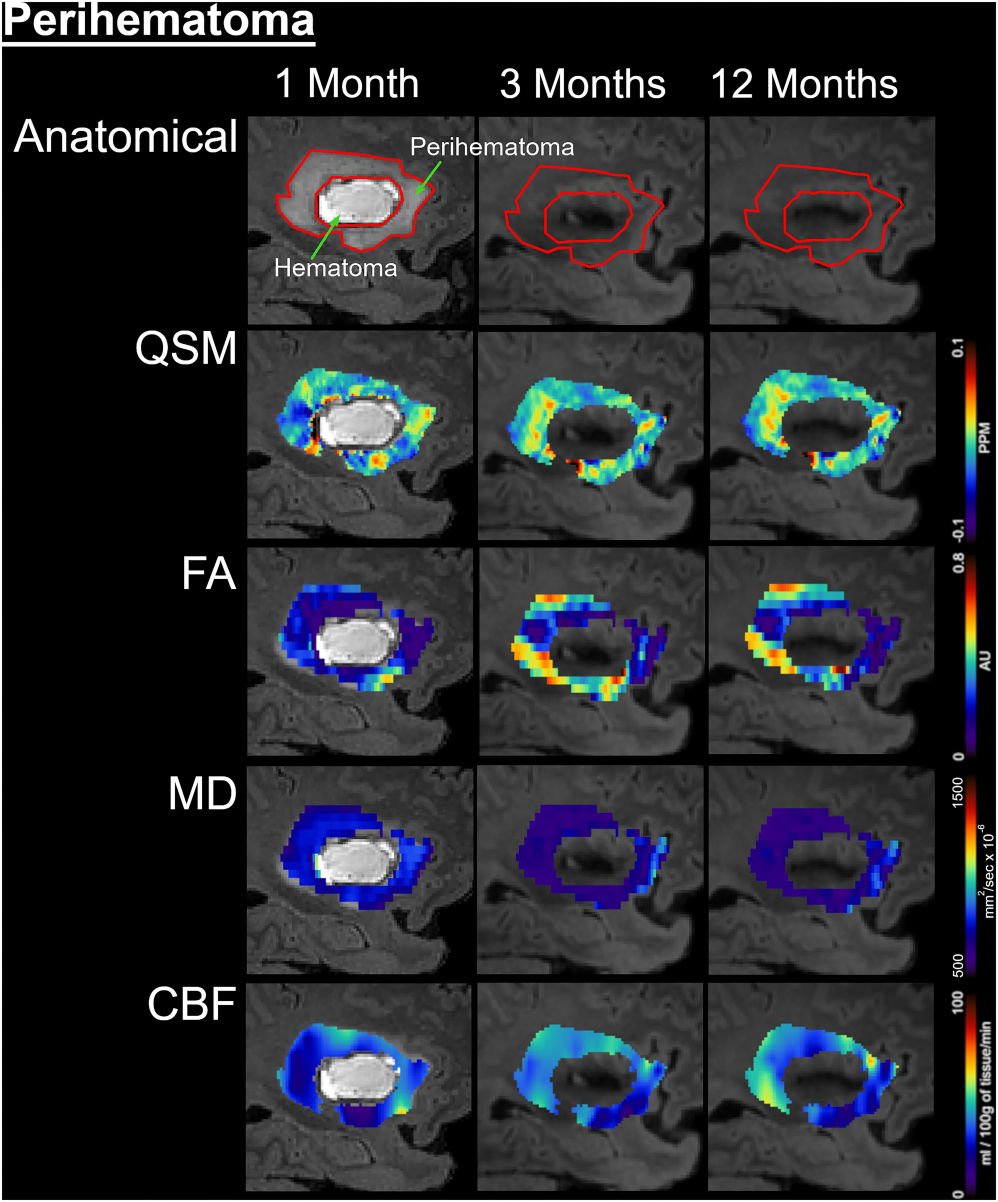
Temporal changes in the perihematomal tissues are shown on a fluid-attenuated inversion recovery (FLAIR) by color-coded voxel by voxel quantitative maps in a represented patient. The rows are illustrating the imaging measurements whereas columns are showing changes at 1, 3, and 12 months of onset. In the top row, the first image outlined the perihematomal and hematoma regions that were used as regions-of-interest (ROI) to measure imaging matrices over time. The rows 2–5 are displaying quantitative susceptibilities maps (QSM), fractional anisotropy (FA), mean diffusivity (MD), and cerebral blood flow (CBF) in the perihematomal tissues overtime, respectively.

**Figure 4:**
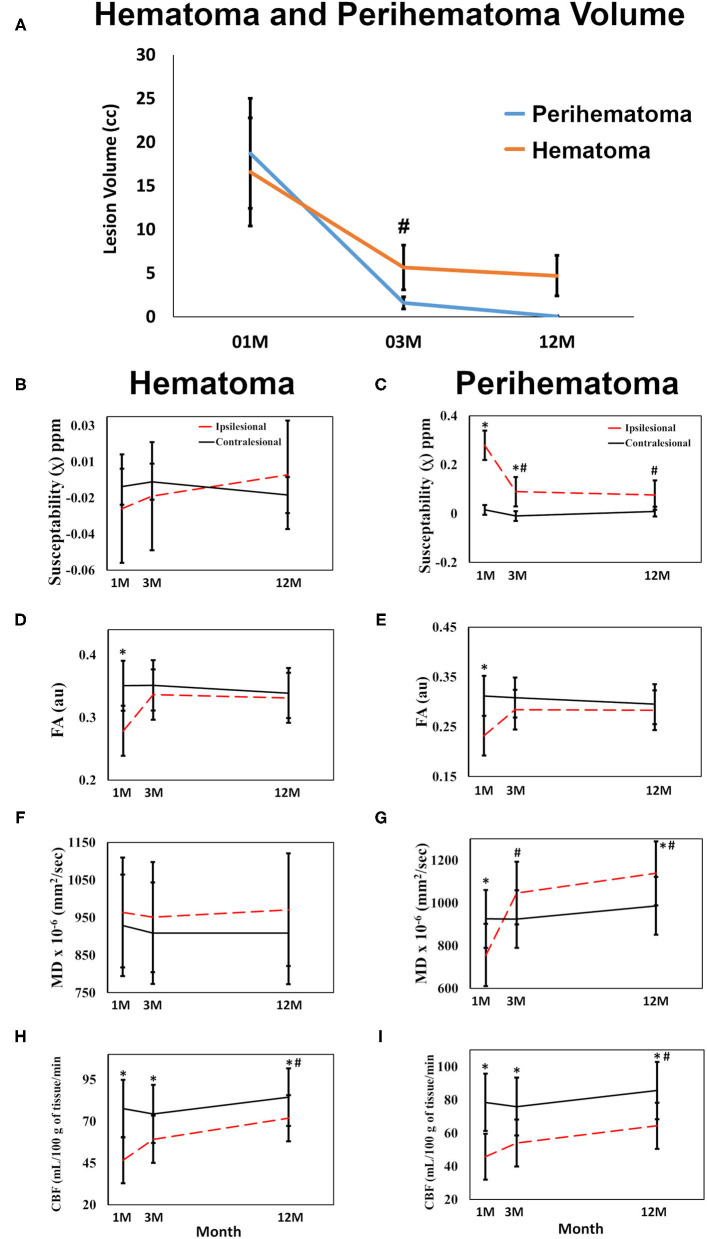
Quantitative changes in imaging matrices are shown over time in the hematomal and perihematomal ROIs compared to the matching contralesional one. **(A)** Significant reduction in both the perihematoma and hematoma volume between 1 and 3 M with no significant reduction thereafter. Plots **(B,C)** changes in susceptibilities; **(D,E)** FA; **(F,G)** MD changes over time in the hematoma and perihematomal ROIs compared to the contralesional side. **(H,I)** temporal changes CBF in the hematoma and perihematoma ROIs as compared to the contralesional side. *p < 0.05 as compared with the contralesional tissues, ^#^*p* < 0.05 ipsilesional changes over time as compared with the 1 M, error bar are STD err.

Figure 1 delineates serial hematoma and perihematomal regions used to calculate the hematoma and perihematomal volume. Both hematoma and perihematomal volumes were significantly (*p* < 0.05) decreased within 3 M of onset. As compared with 1 M, the average hematoma volume (1 M, 16.59 ± 21.03; 3 M, 5.66 ± 8.64 cm^3^) and perihematomal volume (1 M, 18.7 ± 21.8; 3 M, 1.59 ± 2.34 cm^3^) were decreased by 66 and 91.4%, at 3 M, respectively. Between 3 and 12 M, only 16% of the hematoma volume (3 M, 5.66 ± 8.84; 12 M, 4.71 ± 7.70 cm^3^) and 96.2% of perihematomal volume (3 M, 1.59 ± 2.34; 12 M, 0.06 ± 0.12 cm^3^) had resolved. The temporal hematoma and perihematomal volume changes are summarized in **Figure 4A**. Figures 2, 3 (top row) show the typical hematoma and perihematomal region at each time point which was used to calculate changes in the imaging matrices.

### Magnetic Susceptibility

The average hematoma susceptibilities at 1 and 3 M (1 M, 0.274 ± 0.3; 3 M, 0.089 ± 0.03 ppm) were significantly higher (*p* < 0.01) than matching contralesional ones (1 M, 0.021 ± 0.01; 3 M, 0.01 ± 0.01 ppm) whereas no significant difference in susceptibilities was found at 12 M. As compared to 1 M, the hematoma susceptibilities were significantly decreased (*p* < 0.01) at 3 and 12 M. The perihematoma susceptibility at 1 M (−0.029 ± 0.03 ppm) was decreased as compared to the contralesional tissues (−0.008 ± 0.01 ppm). Temporally, perihematomal susceptibilities increased to 0.002 ± 0.03 ppm at 12 M. There was no significant difference in susceptibility between the perihematomal area and the corresponding contralesional tissues. Temporally, perihematomal susceptibility was not significantly changed (Figures 2, 3, row 2; Figures 4B,C).

### Fractional Anisotropy and Mean Diffusivity

FA at 1 M in both the hematoma (0.23 ± 0.03) and perihematomal (0.28 ± 0.02) tissues was significantly decreased (*p* < 0.05) as compared to the contralesional tissues (0.35 ± 0.02 and 0.32 ± 0.02, respectively). Between 1 and 3 M, FA in the hematoma and perihematomal increased to 0.28 ± 0.03 and 0.34 ± 0.02, respectively, but the increase was not statistically significant. Between 3 and 12 M, there was no temporal change in FA in either hematoma or perihematoma. The contralesional FA remained constant throughout the study (Figures 2, 3, row 4; Figures 4D,E). The MD in the hematoma (756 ± 73 x 10^−6^ mm^2^/s) was significantly (*p* < 0.01) decreased as compared to the contralesional area (925 ± 67 x 10^−6^ mm^2^/s) at 1 M. Moreover, the hematomal MD was significantly (*p* < 0.01) higher at 3 and 12 M as compared to 1 Mmonths. Matching contralesional tissue remained constant throughout the study. The average perihematomal MD was slightly higher than the contralesional one and remained elevated throughout the study, but without being statistically different. There was no temporal difference in perihematomal MD (Figures 2, 3, row 4; Figures 4F,G).

### Cerebral Blood Flow

The CBF in both the hematoma and perihematomal regions was significantly decreased (*p* < 0.01) as compared to the matching contralesional tissues at all-time points. Temporally, CBF in both the hematoma (1 M, 45.7 ± 6.9; 12 M, 64.3 ± 6.8 ml/100 g of tissue/min) and perihematomal space (1 M, 46.6 ± 6.9; 12 M, 71.8 ± 6.8 ml/100 g of tissue/min) significantly increased (*p* < 0.05) between 1 and 12 M. There was no change at the contralesional side (Figures 2, 3, row 5; Figures 4H,I).

### Correlation Between Imaging Matrices and Clinical Assessment

Both hematoma and perihematomal volume was significantly associated (*p* < 0.01) with initial but not later mRS scores. A negative association between perihematomal volume and FA (*p* < 0.01) and hematoma volume with CBF (*p* < 0.05) was found in that particular ROI. The susceptibilities in the perihematoma were significantly (*p* < 0.01) correlated with the NIHSS. There was no significant association between clinical assessments and imaging matrices over time. All the correlations between the imaging matrices and clinical scores are summarized in Table 2.

**Table 2:** Summarized association between imaging measurements within hematoma and perihematomal volumes and clinical scores.

	**Perihematoma volume**		**Hematoma volume**		**NIHSS**		**mRS**	
	**β(SE)**	***p*-value**	**β(SE)**	***p*-value**	**β(SE)**	***p*-value**	**β(SE)**	***p*-value**
**Perihematoma**								
χ	−0.001(<0.001)	0.089	–	–	−0.011(0.003)	0.004^a^	0.003(0.006)	0.612
FA	−0.002(0.001)	0.002^a^	–	–	0.000(0.006)	0.981	0.002(0.012)	0.880
MD	4.890(2.470)	0.057	–	–	−4.692(21.353)	0.828	−16.370(39.931)	0.685
CBF	−0.383(0.236)	0.116	–	–	−2.441(2.014)	0.236	−5.954(4.011)	0.149
**Hematoma**								
χ	–	–	0.004(0.003)	0.206	−0.006(0.020)	0.778	−0.006(0.032)	0.852
FA	–	–	−0.002(0.001)	0.083	−0.003(0.008)	0.697	0.011(0.015)	0.462
MD	–	–	−7.231(5.920)	0.236	−16.449(35.142)	0.645	19.608(65.352)	0.767
CBF	–	–	−0.885(0.350)	0.021^a^	1.734(2.212)	0.443	−3.869(3.978)	0.344

*NIHSS, National Institute of Health Stroke Scale; mRS, Modified ranking scale; β(SE), Estimated coefficient from mixed-effects model (Standard Error); χ, Iron induced susceptibilities; FA, Fractional Anisotropy; MD, Mean Diffusivity; CBF, Cerebral Blood Flow*.

a *statistically significant at 0.05 level*.

## Discussion

In this multimodal, serial quantitative MRI pilot study, we assessed long-term microstructural and physiological changes in the perihematomal and hematomal tissues of ICH patients. Hematoma and perihematomal volumes are a strong prognostic tool for patients with acute ICH; however, we have a poor understanding of the long-term changes in the brain after the initial injury. In addition, neurological impairments persist months to years afterward, despite a significant reduction in hematoma and perihematomal volume.

Acute evaluation of the pathological changes in intracerebral hematomas is challenging with MRI. The red blood cell lysis changes the heme iron oxidation state resulting in oxyhemoglobin changing to deoxyhemoglobin, which further converts to methemoglobin, which then leads to hemosiderin. These molecular changes affect MR contrast, making it very challenging to accurately measure hematoma volume in the acute to subacute phase (28). In this study, we add pilot quantitative information on localized changes within the hematoma and perihematomal tissue between the sub-acute to the chronic phase. The GRE MR imaging sequence is considered the gold standard for ICH diagnosis and hematoma volume measurements, but it is known for overestimating the hematoma volume due to blooming artifacts. In addition, it relies on image acquisition protocol making it difficult to reliably estimate the hematoma volume. Susceptibility-weighted imaging (SWI) maximizes the susceptibility sensitivity by using a high-pass filter of phase image, creating a mask to the magnitude image, long echo time, and 3D flow compensated gradient to overcome the artifact. However, both GRE and SWI rely on echo time, SWI is qualitative and encounters artifact due to dipole effect of phase signal. QSM computes susceptibility distribution in the phase component of MR signal. The variation in susceptibility leads to a change in phase signal which can be calculated (23). The serial hematoma volume measurements showed that the hematoma did not fully resolve at 12 M, given the detection of positive susceptibility due to the presence of aggregated paramagnetic iron molecules in the form of hemosiderin. Previously, in a serial study, Sun et al. showed a direct correlation between CT and QSM hematoma volume measurements (29). Concordant with the previous report, our data also show a decrease in hematoma susceptibility between the subacute (1 M) to the chronic stage (3 M) (29, 30). In the perihematomal region, the decrease in negative susceptibility at 1 M suggests an increase in diamagnetic substances, such as water, protein, cerebrospinal fluid (CSF) etc., as compared to the contralesional side. Several animal studies reported diffused iron toxicity to the neighboring perihematomal tissues (31). Contrary to the literature, our data show a decrease in susceptibilities, suggesting either there was no diffused iron in the surrounding tissues or our methods lacked the required sensitivity. Another possible explanation is an increase in diamagnetic water molecules, which could be diluting the iron content in the perihematomal area. The minimal reduction in hematoma volume and susceptibilities between 3 and 12 M suggest that iron remains localized and no significant reduction in volume occurred beyond 3 M. Analogous to animal studies (32), QSM data also showed signal intensity heterogeneity within the hematoma, suggesting the non-uniform distribution of aggregated free iron. This signal heterogeneity could be due to the restricted movement of iron molecules accumulating in certain regions of the hematoma, suggesting the presence of intact tissue (33). This heterogeneity within hematoma volume creates variation in the MRI signal; therefore, very high standard deviations in the χ measurement were found.

As compared to the contralesional tissues, both the hematoma and perihematomal regions exhibited a reduction in FA at 1 M, indicating an increase in isotropic diffusion, indicative of changes in microstructural tissue architecture. The decrease in perihematomal FA is in line with previously published reports in acute and subacute stages (34). However, both regions showed a FA regain at 12 M, suggesting reversible changes. We postulate that the FA changes in the hematoma and perihematomal ROIs could be the results of two different molecular phenomena which can be supported by the mean diffusivity changes. Mathematically, FA and MD are inversely related as shown in the perihematomal regions where ipsilesional FA is relatively lower, and MD is higher than at the contralesional side. However, this is not the case in the hematoma region where both FA and MD decreased at 1 M as compared to the contralesional side. We speculate the MD decrease in the hematoma at 1 M is due to the presence of cellular debris in the region which restricts the movement of water molecules in the area. Over time, the debris is cleared resulting in an increase in MD in the hematomal region. Furthermore, a continual increase in MD in the hematomal region suggests possible tissue loss, and increasing regional extracellular fluid or possibly CSF, which will be verified by measuring longitudinal relaxation time in future studies. A serial study by Knight et al. reported a similar pattern of change in MD within hematoma over 3 months (35). An increase of FA in both the perihematomal and hematoma regions between the first two imaging time points suggests an increase in restricted diffusion, which could be due to microstructural restoration or an increase in regional cellular debris. However, a negative association between perihematomal (*p* < 0.01) and hematoma (*p* = 0.083) volume with FA is most likely due to improvement in microstructural integrity. No change in FA between 3 and 12 months suggests there is no microstructural repair beyond 3 M.

Previously, both human and animal studies have linked mechanical tissue compression by the hematoma and perihematoma to compromise capillary blood flow and possibly the creation of ischemic environments in the acute setting of ICH (36–38). As compared to the contralesional ROI, our data showed a significant decrease in CBF in both regions. Several previous studies reported post-ICH perihematomal CBF declines between acute to sub-chronic stages (39–42). The temporal reduction in CBF probably occurred before our first measurement, suggesting the decrease in CBF occurred in the acute phase or it may vary with ICH location, size, and amount of tissue pressure by the expansion or it could be a result of aggressive hypertension management in the acute phase (43). However, our data also showed a gradual increase in CBF in both regions starting at 1 M after injury, suggesting a change in supply and demand which could be due to cellular function. The non-uniform increase in the CBF maps in each region suggests that not all tissues within the perihematoma or hematoma were damaged. The rise in CBF may also reflect tissue repair or capillary sprouting by reorganization over time.

We identified imaging characteristics that correlated with clinical scores in this study. However, the NIHSS and mRS may not be appropriate to detect clinical changes over 1 year. Using domain-specific endpoints may be more appropriate in further studies. Another interesting observation was that three out of four patients with acute midline-shift developed Wallerian degeneration. The MRI findings of WD depend on the stage of the axonal injury process. In the first few weeks after the brain insult, no appreciable imaging abnormalities can be seen; however, as early as 5 weeks after the injury, the white matter (WM) tracts arising from the injured brain tissue demonstrate a slightly increased T1w signal and low T2w signal due to accelerated axonal breakdown with relative sparing of the lipid-containing structures. The imaging findings of WD become more evident after 3 months after the insult when the T1w signal intensity drops, and the T2w-based sequences show a significant increase in signal intensity as a reflection of myelin lipid breakdown and settling gliosis. These findings ultimately progress into volume loss, which is easily picked up visually by comparing symmetry.

Our study has a number of limitations. As a pilot study, the sample size was small with wide variability in hematoma size and location. On the other hand, this study provides detailed and repeated assessments on a number of imaging endpoints over 1 year, not been reported previously. We found important, dynamic, structural, and physiological changes occur over time within the hematoma and perihematomal areas. The changes may have important clinical implications to better understand the chronic and persistent neurological impairments of patients with ICH. A lack of repair in the chronic setting may underlie persistent impairments. The findings help to build new hypotheses and thus merit larger studies to identify extended treatment time windows or specific therapies that could modify these changes in the subacute to chronic phase to ultimately promote better recovery of specific clinical impairments in ICH patients. Finally, since most of our patient population had a hypertensive etiology, we will also need to study the generalizability of our findings and assess differential long-term changes based on ICH etiology.

In conclusion, we present novel data on the longitudinal changes that occur within the hematoma and perihematomal areas in the brain over 1 year in patients with ICH. We find active, persistent, and long-term microstructural and physiological changes that require further research in larger observational studies.

## Data Availability Statement

The raw data supporting the conclusions of this article will be made available by the authors, without undue reservation.

## Ethics Statement

The studies involving human participants were reviewed and approved by Institutional Review Board of the University of Texas Health Sciences Center at Houston and by the Memorial Hermann Hospital Office of Research. The patients/participants provided their written informed consent to participate in this study.

## Author Contributions

MH: concept and design, data analysis and interpretation, and writing manuscript. SB: data analysis and created figures. OA: radiological assessment and data analysis. RG: statistical analyses. SG and MP: clinical and neurological assessment. XZ and JA: manuscript writing. SS: financial support, data interpretation, writing, and final approval of the manuscript. All authors contributed to the article and approved the submitted version.

## Conflict of Interest

The authors declare that the research was conducted in the absence of any commercial or financial relationships that could be construed as a potential conflict of interest.

## Publisher's Note

All claims expressed in this article are solely those of the authors and do not necessarily represent those of their affiliated organizations, or those of the publisher, the editors and the reviewers. Any product that may be evaluated in this article, or claim that may be made by its manufacturer, is not guaranteed or endorsed by the publisher.
